# Association between the atherogenic index of plasma and prehypertension or hypertension among adults in Fujian, China: a population-based cross-sectional study

**DOI:** 10.3389/fcvm.2026.1708190

**Published:** 2026-02-05

**Authors:** Yijun Jiang, Danjing Chen, Jingru Huang, Huajing Chang, He Zhang, Yongfeng Cai, Hua Fang, Ying Han, Xian-E Peng

**Affiliations:** 1 Department of Epidemiology and Health Statistics, Fujian Provincial Key Laboratory of Environment Factors and Cancer, School of Public Health, Fujian Medical University, Fuzhou, Fujian, China; 2 College of Integrated Chinese and Western Medicine, Fujian University of Traditional Chinese Medicine, Fuzhou, Fujian, China; 3 Department of Geriatrics, The First Affiliated Hospital of Fujian Medical University, Fuzhou, China; 4 Fujian Hypertension Research Institute, The First Affiliated Hospital of Fujian Medical University, Fuzhou, China; 5 Branch of National Clinical Research Center for Aging and Medicine, The First Affiliated Hospital of Fujian Medical University, Fuzhou, Fujian, China

**Keywords:** atherogenic index of plasma, blood pressure, cross-sectional study, hypertension, prehypertension

## Abstract

**Background:**

Elevated blood pressure may share a similar pathophysiology with cardiovascular disease (CVD). Although the atherogenic index of plasma (AIP) has been associated with hypertension and CVD, evidence on the association between the AIP and prehypertension risk remains limited. This study aims to explore the relationship of the AIP with the risks of prehypertension and hypertension in a Chinese population.

**Methods:**

This study was based on a population-based cross-sectional survey data of Fujian province and was conducted between August 2020 and April 2021. The association between the AIP and prehypertension/hypertension risk was assessed through multivariate logistic regression. Four-knot restricted cubic splines (RCS) were performed to examine the potential dose–response relationships. Subgroup analyses were used to assess the heterogeneity of the associations across different population subgroups, and an receiver operating characteristic (ROC) curve analysis was used to determine the cutoff values for predicting prehypertension/hypertension. The mediating roles of the two types of inflammatory indicators were analyzed through mediation analysis. Several sensitivity analyses were also conducted to further assess the robustness of the results.

**Results:**

A total of 9,473 adults were included in the final analysis, including 3,273 diagnosed with prehypertension and 3,248 with hypertension. Overall, participants with elevated AIP levels demonstrated higher prehypertension detection rates and hypertension prevalence (all *P* < 0.05). The multivariate logistic regression model showed that the AIP was significantly positively associated with prehypertension [per 0.1-unit increment in the AIP: odds ratio (OR), 1.05, 95% confidence interval (CI): 1.03–1.07] and hypertension (per 0.1-unit increment in the AIP: OR, 1.11, 95% CI: 1.09–1.14), respectively. Compared with the lowest quartile of the AIP, participants in the highest quartile had a higher risk of prehypertension [OR: 1.58 (1.32, 1.90)] and hypertension [OR: 2.30 (1.86, 2.84)]. No evidence of a non-linear association was observed between the AIP and the risk of prehypertension (*P*_non-linea*r*_ = 0.178) or hypertension (*P*_non-linea*r*_ = 0.087). There were significant interactions between the AIP and both residential location and educational level regarding the risk of blood pressure classification (*P*_interaction_ < 0.05). Furthermore, an ROC analysis indicated a higher clinical predictive value of the AIP for hypertension (AUC = 0.721, 95% CI: 0.708–0.734). White blood cell count mediated 10.91% and 15.52% of the total effect of the AIP on prehypertension and hypertension, respectively.

**Conclusion:**

The AIP is positively associated with the risk of prehypertension and hypertension among participants ≥ 18 years of age in China, with white blood cell count potentially mediating a part of this association, which indicates that monitoring and maintaining optimal AIP levels may help prevent the deterioration of blood pressure categories.

## Introduction

Cardiovascular disease (CVD), a group of disorders affecting the heart and blood vessels, is one of the leading causes of mortality worldwide (1). The development of CVD is closely related to abnormalities in blood pressure regulation and metabolic homeostasis, which often coexist with dyslipidemia, diabetes, obesity, and unhealthy lifestyle behaviors (2). Among these, hypertension stands out as one of the most prevalent and influential independent risk factors for CVD, posing a major public health challenge (3). According to data from the World Health Organization (WHO), hypertension affects 33% of adults aged 30–79 years globally (4) and has been confirmed to be associated with increased mortality risk from cardiovascular and renal diseases (5–7). In addition, approximately 30%–50% of adults worldwide are in the prehypertensive stage (8, 9), and individuals in this group face two to three times the risk of developing hypertension compared with those with normal blood pressure (10). Notably, even systolic blood pressure (SBP) within the 115–130 mmHg is associated with elevated mortality risk (11–13). Had all adults worldwide maintained systolic blood pressure at the estimated theoretical minimum-risk level (110–115 mmHg) in 2019, approximately 19% of global deaths that year could have been prevented (12, 14). According to a China hypertension survey, 23.2% (approximately 244.5 million) of the Chinese adult population have hypertension, and an additional 41.3% (approximately 435.3 million) are diagnosed with prehypertension (9). Therefore, early intervention in hypertension, especially prehypertension, is critical for preventing or delaying the onset of cardiovascular diseases and their complications.

The pathological mechanisms underlying blood pressure regulation are complex (15–17). Recent studies highlight the central role of metabolic disorders in blood pressure modulation, with atherogenic lipid abnormalities promoting vascular remodeling through oxidative stress and endothelial dysfunction (18–20). In addition, inflammation represents an important mechanism linking atherosclerosis and hypertension, as it can exacerbate vascular injury and thereby contribute to the onset and progression of hypertension (21–23). The atherogenic index of plasma (AIP), a biomarker integrating the ratio of triglycerides to high-density lipoprotein cholesterol (TG/HDL-C), has received extensive attention from researchers in recent years. Previous studies have demonstrated that the AIP is a comprehensive index for assessing the risk of metabolic disorders, CVD (24, 25), and type 2 diabetes (T2DM) (26, 27).

Multiple cross-sectional and longitudinal studies across diverse populations have demonstrated that higher AIP levels are significantly and positively associated with both the prevalence and the incidence of hypertension (28–39). Notably, this association appears to exhibit population heterogeneity, with some studies reporting stronger associations in middle-aged individuals (40–64 years) and more pronounced correlations in women than in men (29, 35, 37–39). Therefore, the influence of population heterogeneity on the AIP–blood pressure relationship still requires a stratified analysis. Moreover, most existing studies treat blood pressure status as a binary outcome, thereby overlooking the potential risk gradient of the AIP across the continuum from normotension, through prehypertension, to hypertension. Comprehensive investigations into the relationship between the AIP and prehypertension, a critical stage for early intervention with maximal health benefits, are limited (38, 39). Therefore, we conducted a population-based cross-sectional study to explore the association of the AIP with the risk of prehypertension and hypertension in a Chinese population and to further explore the mediating role of inflammation in this relationship.

## Methods

### Study design and population

**Figure 1:**
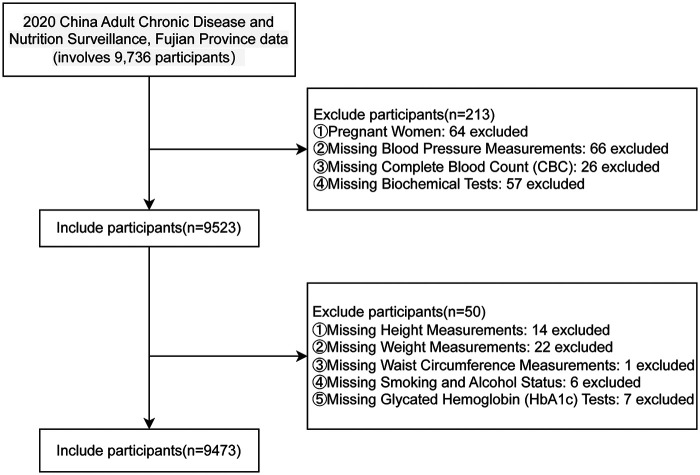
A flow diagram of study subjects included in the analysis.

The data were obtained from the Fujian Provincial Surveillance Site of the China Cardiovascular Disease and Risk Factors Surveillance Program, a cross-sectional study jointly conducted by the National Center for Cardiovascular Diseases and the First Affiliated Hospital of Fujian Medical University from August 2020 to April 2021. This study enrolled permanent residents aged ≥18 years with ≥6 months of local residency in Fujian Province, China. A stratified multistage random sampling method was employed to select a provincially representative sample for assessing the prevalence of major cardiovascular diseases. All participant information was systematically collected through standardized face-to-face surveys by well-trained examiners, comprising three principal components: questionnaire surveys, physical examinations, and laboratory assessments. Laboratory tests included the collection of fasting blood and urine specimens for the measurement of multiple indicators. After cryopreservation and cold-chain transportation, complete blood count was tested locally, while the remaining biochemical indicators were uniformly tested in a designated laboratory to ensure comparability. Specifically, this study utilized comprehensive data from 9,736 individuals in the Fujian Provincial Surveillance Site during the 2020–2021 period. Following a rigorous screening process, 9,473 participants met the stringent inclusion criteria for final analysis. Figure 1 presents a detailed flowchart outlining the participant selection procedure for this study.

### Data collection

As described by previous literature (40), participants completed a standardized questionnaire developed by the National Coordinating Center of Fu Wai Hospital (Beijing, China) through face-to-face interviews conducted by trained staff. The questionnaire covered sociodemographic factors (e.g., gender, age, occupation, education, marital status, place of residence, economic status, and health insurance), behavioral factors (e.g., smoking, alcohol consumption, physical activity, and regular sleep), personal medical history (e.g., hypertension, diabetes, dyslipidemia, and hyperuricemia), and family history of hypertension. The physical examination included measurements of height, weight, waist circumference, and blood pressure. To reduce information bias, the data collection procedures were carried out independently, and investigators conducting questionnaires, blood pressure measurements, and laboratory assays were blinded to one another's data as well as to the study outcomes.

### Definitions of prehypertension and hypertension

Blood pressure was measured using the OMRON HBP-1300 Professional Portable Blood Pressure Monitor (OMRON, Kyoto, Japan) three times on the right arm positioned at the heart level after the participant was sitting at rest for 5 min, with 30 s between each measurement with an observer present. Valid measurements were identified as those in which two out of three SBP and DBP readings differed by  ≤10 mmHg, and the average of the three readings was used for analysis. According to the Chinese Guidelines for the Prevention and Treatment of Hypertension (2024 Revision) (41), hypertension was defined as systolic blood pressure (SBP) ≥ 140 mmHg and/or diastolic blood pressure (DBP) ≥ 90 mmHg and/or a documented history of hypertension with antihypertensive medication use within the preceding two weeks. Prehypertension was classified as SBP ranging from 120 to 139 mmHg and/or DBP ranging from 80 to 89 mmHg, while normal blood pressure was defined as SBP <120 mmHg and DBP <80 mmHg.

### AIP calculation

The AIP was calculated as the logarithmic transformation of the ratio of TG to HDL-C, expressed as AIP = log(TG/HDL-C) (42). Because of its small magnitude, the AIP was multiplied by 10 (AIP  ×  10) when calculating the odds ratios (ORs) for continuous variables in this study (43).

### Potential covariates

In this study, potential covariates were selected *a priori* based on existing literature and clinical expertise to comprehensively evaluate their potential influence on the study outcomes. These covariates included sociodemographic characteristics, anthropometric indicators, lifestyle behaviors, and laboratory parameters. Sociodemographic characteristics included sex (male or female), age groups (≥65, 40–64, and <40 years), residential location (urban or rural), education level (no schooling, primary school, junior high school, senior high school or technical school, and college or above), marital status (unmarried, married/remarried/cohabiting, separated, divorced, or widowed), and per capita annual household income (<10,000; 10,000–20,000; 20,000–30,000; 30,000–50,000; 50,000–100,000; or ≥100,000 yuan). Anthropometric indicators included body mass index (BMI), calculated as weight (kg) divided by height squared (m^2^), and categorized as underweight (<18.5), normal weight (18.5–23.9), and overweight (24.0–27.9), or obese (≥28.0). Waist circumference (WC) was measured horizontally at the midpoint between the iliac crest and the 12th rib along the midaxillary line, recorded to the nearest 0.1 cm at the end of expiration. A WC of <85 cm in men or <80 cm in women was defined as “normal”; 85–89.9 cm in men or 80–84.9 cm in women as “precentral obesity”; and ≥90 cm in men or ≥85 cm in women as “central obesity,” according to the *Expert Consensus on the Prevention and Control of Obesity in Chinese Adults* (44). Lifestyle factors covered sleep duration [good (7–9 h), insufficient (<7 h), or excessive (>9 h)], smoking status (never, former, or current smoker), alcohol consumption [none, daily (≥1 drink per day), weekly (≥1 drink per week), monthly (≥1 drink per month), or rarely (<1 drink per month)], and physical activity level [insufficient (<150 min/week of moderate-to-vigorous physical activity) or sufficient (≥150 min/week)]. Laboratory parameters included glycated hemoglobin (HbA1c), classified as diabetes (≥6.5%), prediabetes (5.7%–6.4%), or normal (<5.7%) (45); fasting triglycerides (TG); total cholesterol (TC); low-density lipoprotein cholesterol (LDL-C); high-density lipoprotein cholesterol (HDL-C); serum creatinine (Cr); and uric acid (UA). Blood biochemical parameters, including HbA1c, TC, TG, HDL-C, LDL-C, Cr, and UA, were measured using a Beckman AU680 analyzer. HbA1c was determined by high-performance liquid chromatography; oxidase methods were used for TC and TG; direct methods were applied for HDL-C and LDL-C; and the enzymatic method was used for Cr and UA.

### Statistical analysis

Based on the quartiles of the AIP, all study participants were categorized into four groups. Normally distributed continuous variables were presented as mean ± standard deviation (SD), with group comparisons made using one-way ANOVA. Non-normally distributed continuous variables were presented as median (interquartile range, IQR), with group comparisons made using the Kruskal–Wallis *H* test. Categorical variables were presented as number (percentage), and group comparisons were performed using the chi-square test. The generalized variance inflation factor (GVIF) was applied to assess multicollinearity among covariates. All variables showed acceptable collinearity, with GVIF^[1/(2 × Df)] values below 2. The proportional odds assumption for ordinal logistic regression was evaluated using the Brant test. Given the violation of this assumption, multinomial logistic regression was applied in subsequent analyses. Three models were utilized in this study: Model 1 was adjusted for sex and age groups; Model 2 was adjusted for Model 1 + sociodemographic factors (residential location, education levels, per capita annual household income, and marital status) and lifestyle factors (sleep status, smoking, alcohol consumption, and physical activity); Model 3 was adjusted for Model 2 + anthropometric measures (BMI groups and WC groups) and laboratory parameters (HbA1c groups, TC, Cr, and UA). The results from the logistic regression analysis were presented as ORs and 95% confidence intervals (CIs). Subgroup analyses were conducted to evaluate potential effect modifications in the associations between the AIP and prehypertension/hypertension by sex, age groups (<40, 40–64, and ≥65 years) (46), BMI groups, smoking, alcohol consumption, residential location, sleep status, and HbA1c groups. Likelihood ratio tests were employed to assess statistical interactions between the AIP and stratification variables.

For further analyses, prehypertension and hypertension were treated as separate binary outcomes. Restricted cubic spline (RCS) analyses with four knots were conducted to explore potential non-linear relationships between the AIP (as a continuous variable) and prehypertension/hypertension risks. Receiver operating characteristic (ROC) curves were generated to identify optimal AIP cutoff values for predicting prehypertension and hypertension, with the optimal thresholds determined using the Youden index. The sensitivity and specificity of these thresholds were calculated, with diagnostic accuracy quantified by the area under the curve (AUC). The mediating roles of the two types of inflammatory indicators [white blood cells (WBCs) and neutrophils (NE)] were analyzed through mediation analysis. In sensitivity analyses, we compared the performance of restricted cubic spline models with different knot specifications based on the Akaike information criterion (AIC), Bayesian information criterion (BIC), likelihood ratio tests, and tests for non-linearity, with the likelihood ratio test given priority. This approach was adopted to mitigate potential bias arising from arbitrary knot placement. In addition, we evaluated the potential impact of unmeasured confounders on the mediation effect by calculating the sensitivity parameter *ρ*, which represents the correlation between the residuals of the mediator and the outcome models. We also reported R^2^_M*R^2^_Y* (the proportion of variance in the mediator and outcome jointly explained by using a hypothetical unmeasured confounder) and R^2^_M∼R^2^_Y∼ (the proportion of residual variance explained by using such a confounder after controlling for covariates). All statistical tests were two-tailed, with *P* < 0.05 considered statistically significant. Analyses were implemented using R software (version 4.4.2).

## Results

### Characteristics of participants

Table 1 presents the demographic and clinical characteristics of participants stratified by AIP quartiles. A total of 9,473 adults were included in the final analysis, comprising 4,697 males (49.6%) and 4,776 females (50.4%). The median age (±SD) was 48.51 ± 18.42 years. Of the 9,473 participants, 3,273 (34.55%) had prehypertension and 3,248 (34.28%) had hypertension. Except the variables of physical activity levels and annual household income per capita, all variables demonstrated statistically significant differences among AIP groups (Q1–Q4). Compared with the other groups, the participants in the AIP Q4 group were often male, older, and urban residents, had lower education levels, were current smokers and alcohol drinkers, had obesity and diabetes mellitus, and had higher levels of BMI, WC, SBP, DBP, HbA1c, Cr, and UA (all *P* < 0.05). Conversely, HDL-C was higher in the Q1 group and showed a negative association with the AIP (*P* < 0.001). Importantly, a higher proportion of individuals with prehypertension or hypertension were observed in the AIP Q4 group compared with the lower quartile AIP groups (*P* < 0.001).

**Table 1:** Baseline characteristics of participants according to the change in the AIP.

Variable	Overall	Quartiles of the AIP				
		Q1 [−0.7360, 0.0354]	Q2 [0.0354, 0.2460]	Q3 [0.2461, 0.4780]	Q4 [0.4781, 2.0200]	*P*-value
Included participants	9,473	2,371	2,366	2,368	2,368	
Sex, *n* (%)						<0.001
Male	4,697 (49.6)	784 (33.1)	1,029 (43.5)	1,280 (54.1)	1,604 (67.7)	
Female	4,776 (50.4)	1,587 (66.9)	1,337 (56.5)	1,088 (45.9)	764 (32.3)	
Age (years)	44.62 ± 18.04	39.16 ± 17.67	44.17 ± 18.70	47.66 ± 18.31	47.48 ± 16.05	<0.001
Age groups, *n* (%)						<0.001
<40 years	4,279 (45.2)	1,407 (59.3)	1,089 (46.0)	915 (38.6)	868 (36.7)	
40–64 years	3,640 (38.4)	688 (29.0)	879 (37.2)	961 (40.6)	1,112 (47.0)	
≥65 years	1,554 (16.4)	276 (11.6)	398 (16.8)	492 (20.8)	388 (16.4)	
Residential location, *n* (%)						<0.001
Urban	3,515 (37.1)	772 (32.6)	864 (36.5)	882 (37.2)	997 (42.1)	
Rural	5,958 (62.9)	1,599 (67.4)	1,502 (63.5)	1,486 (62.8)	1,371 (57.9)	
Marital status, *n* (%)						<0.001
Unmarried	2,133 (22.5)	783 (33.0)	597 (25.2)	412 (17.4)	341 (14.4)	
Married/remarried/cohabiting	6,790 (71.7)	1,490 (62.8)	1,616 (68.3)	1,796 (75.8)	1,888 (79.7)	
Separated	20 (0.2)	6 (0.3)	6 (0.3)	5 (0.2)	3 (0.1)	
Divorced	152 (1.6)	36 (1.5)	30 (1.3)	41 (1.7)	45 (1.9)	
Widowed	378 (4.0)	56 (2.4)	117 (4.9)	114 (4.8)	91 (3.8)	
Education levels, *n* (%)						<0.001
No schooling	1,178 (12.4)	248 (10.5)	326 (13.8)	326 (13.8)	278 (11.7)	
Primary school	1,649 (17.4)	350 (14.8)	402 (17.0)	479 (20.2)	418 (17.7)	
Junior high school	2,212 (23.4)	465 (19.6)	508 (21.5)	562 (23.7)	677 (28.6)	
Senior high school/technical school	1,759 (18.6)	461 (19.4)	422 (17.8)	436 (18.4)	440 (18.6)	
College or above	2,675 (28.2)	847 (35.7)	708 (29.9)	565 (23.9)	555 (23.4)	
Per capita annual household income, *n* (%)						0.059
<10,000 RMB	1,140 (12.0)	275 (11.6)	307 (13.0)	317 (13.4)	241 (10.2)	
10,000–20,000 RMB	2,066 (21.8)	509 (21.5)	533 (22.5)	526 (22.2)	498 (21.0)	
20,000–30,000 RMB	2,150 (22.7)	541 (22.8)	526 (22.2)	532 (22.5)	551 (23.3)	
30,000–50,000 RMB	1,848 (19.5)	480 (20.2)	460 (19.4)	427 (18.0)	481 (20.3)	
50,000–100,000 RMB	1,472 (15.5)	372 (15.7)	354 (15.0)	351 (14.8)	395 (16.7)	
≥100,000 RMB	797 (8.4)	194 (8.2)	186 (7.9)	215 (9.1)	202 (8.5)	
Smoking, *n* (%)						<0.001
None	6,961 (73.5)	2,021 (85.2)	1,877 (79.3)	1,642 (69.3)	1,421 (60.0)	
Former	338 (3.6)	52 (2.2)	78 (3.3)	95 (4.0)	113 (4.8)	
Current	2,174 (22.9)	298 (12.6)	411 (17.4)	631 (26.6)	834 (35.2)	
Alcohol consumption, *n* (%)						<0.001
None	6,408 (67.6)	1,756 (74.1)	1,704 (72.0)	1,569 (66.3)	1,379 (58.2)	
Rarely	1,077 (11.4)	274 (11.6)	270 (11.4)	272 (11.5)	261 (11.0)	
Monthly	880 (9.3)	169 (7.1)	186 (7.9)	247 (10.4)	278 (11.7)	
Weekly	786 (8.3)	108 (4.6)	146 (6.2)	193 (8.2)	339 (14.3)	
Daily	322 (3.4)	64 (2.7)	60 (2.5)	87 (3.7)	111 (4.7)	
Physical activity, *n* (%)						0.319
Insufficient	2,260 (23.9)	540 (22.8)	564 (23.8)	562 (23.7)	594 (25.1)	
Sufficient	7,213 (76.1)	1,831 (77.2)	1,802 (76.2)	1,806 (76.3)	1,774 (74.9)	
Sleep status, *n* (%)						
Good	6,198 (65.4)	1,607 (67.8)	1,552 (65.6)	1,501 (63.4)	1,538 (64.9)	0.012
Insufficient	2,599 (27.4)	589 (24.8)	636 (26.9)	699 (29.5)	675 (28.5)	
Excessive	676 (7.1)	175 (7.4)	178 (7.5)	168 (7.1)	155 (6.5)	
Height (cm)	161.46 ± 9.04	160.08 ± 8.71	160.79 ± 8.98	161.50 ± 9.20	163.46 ± 8.93	<0.001
Weight (kg)	62.08 ± 12.38	55.55 ± 9.62	59.82 ± 10.95	63.54 ± 11.72	69.42 ± 12.63	<0.001
WC (cm)	79.84 ± 10.91	72.88 ± 9.01	77.85 ± 9.99	81.89 ± 9.79	86.73 ± 9.77	<0.001
WC groups, *n* (%)						
Normal	5,672 (59.9)	2,005 (84.6)	1,583 (66.9)	1,256 (53.0)	828 (35.0)	
Precentral obesity	1,629 (17.2)	202 (8.5)	406 (17.2)	486 (20.5)	535 (22.6)	
Central obesity	2,172 (22.9)	164 (6.9)	377 (15.9)	626 (26.4)	1,005 (42.4)	<0.001
BMI (kg/m^2^)	23.72 ± 3.72	21.64 ± 3.10	23.08 ± 3.42	24.29 ± 3.47	25.88 ± 3.53	<0.001
BMI groups, *n* (%)						<0.001
Underweight	568 (6.0)	318 (13.4)	156 (6.6)	70 (3.0)	24 (1.0)	
Normal	4,816 (50.8)	1,608 (67.8)	1,380 (58.3)	1,116 (47.1)	712 (30.1)	
Overweight	2,952 (31.2)	356 (15.0)	660 (27.9)	879 (37.1)	1,057 (44.6)	
Obese	1,137 (12.0)	89 (3.8)	170 (7.2)	303 (12.8)	575 (24.3)	
HbA1c (%)	5.61 ± 0.78	5.40 ± 0.56	5.52 ± 0.68	5.64 ± 0.71	5.88 ± 1.02	<0.001
HbA1c groups, *n* (%)						<0.001
Normal	6,155 (65.0)	1,894 (79.9)	1,639 (69.3)	1,429 (60.3)	1,193 (50.4)	
Prediabetes	2,706 (28.6)	432 (18.2)	635 (26.8)	781 (33.0)	858 (36.2)	
Diabetes	612 (6.5)	45 (1.9)	92 (3.9)	158 (6.7)	317 (13.4)	
UA (μmol/L）	352.24 ± 93.74	314.49 ± 79.91	337.49 ± 86.08	360.56 ± 88.92	396.45 ± 98.95	<0.001
Cr (μmol/L）	75.48 ± 23.29	70.07 ± 17.80	74.19 ± 21.56	77.45 ± 23.40	80.19 ± 27.99	<0.001
TG (mmol/L）	1.42 ± 1.35	0.57 ± 0.14	0.90 ± 0.18	1.33 ± 0.27	2.89 ± 2.01	<0.001
TC (mmol/L）	4.92 ± 1.04	4.66 ± 0.93	4.79 ± 1.00	5.01 ± 1.03	5.24 ± 1.09	<0.001
HDL-C (mmol/L）	1.41 ± 0.31	1.68 ± 0.29	1.47 ± 0.26	1.33 ± 0.23	1.16 ± 0.20	<0.001
LDL-C (mmol/L）	3.06 ± 0.87	2.75 ± 0.77	3.02 ± 0.84	3.27 ± 0.88	3.22 ± 0.90	<0.001
WBC (1,000 cells/µL)	6.68 ± 5.97	6.05 ± 1.53	6.45 ± 1.70	6.76 ± 1.66	7.44 ± 11.57	<0.001
NE (1,000 cells/µL)	3.85 ± 3.70	3.60 ± 5.88	3.72 ± 1.37	3.89 ± 1.28	4.18 ± 4.04	<0.001
SBP (mmHg)	128.95 ± 20.08	121.20 ± 17.91	127.46 ± 20.35	131.48 ± 19.84	135.69 ± 19.27	<0.001
DBP (mmHg)	80.57 ± 10.91	76.39 ± 9.56	79.23 ± 10.62	81.27 ± 10.41	85.38 ± 11.01	<0.001
Blood pressure categories, *n* (%)						<0.001
Normal	2,952 (31.2)	1,168 (49.3)	837 (35.4)	592 (25.0)	355 (15.0)	
Prehypertension	3,273 (34.6)	755 (31.8)	841 (35.5)	849 (35.9)	828 (35.0)	
Hypertension	3,248 (34.3)	448 (18.9)	688 (29.1)	927 (39.1)	1,185 (50.0)	

AIP, atherogenic index of plasma; RMB, Renminbi; WC, waist circumference; BMI, body mass index; HbA1c, hemoglobin A1c; UA, uric acid; Cr, creatinine; TC, total cholesterol; TG, triglycerides; HDL-C, high-density lipoprotein cholesterol; LDL-C, low-density lipoprotein cholesterol; WBC, white blood cell; NE, Neutrophils; SBP, systolic blood pressure; DBP, diastolic blood pressure.

### Association between AIP and prehypertension/hypertension

Significant positive associations were observed between the AIP and prehypertension or hypertension, irrespective of confounding factor adjustment (Table 2). The results of the multivariable logistic regression Model 3 showed that each 0.1-unit increment in the AIP was associated with a 5% increased risk of prehypertension (OR = 1.05, 95% CI = 1.03–1.07, *P* < 0.001) and an 11% elevated risk of hypertension (OR = 1.11, 95% CI = 1.09–1.14, *P* < 0.001). When the AIP was further categorized into quartiles, it was also associated with the risk of prehypertension or hypertension. Compared with the lowest quartile (Q1), the participants in the higher AIP quartiles (Q2–Q4) demonstrated an increased risk of both prehypertension and hypertension (*P* for trend <0.001). Notably, individuals in the highest AIP quartile (Q4) exhibited a 58% increased risk of prehypertension (OR_Q4 VS. Q1_ = 1.58, 95% CI = 1.32–1.90, *P* < 0.001) and a 152% higher risk of hypertension (OR_Q4 VS. Q1_ = 2.30, 95% CI = 1.86–2.84, *P* < 0.001).

**Table 2:** Association between the AIP and prehypertension/hypertension.

Variable	Unadjusted model OR (95% CI)	*P*-value	Model 1 OR (95% CI)	*P*-value	Model 2 OR (95% CI)	*P*-value	Model 3 OR (95% CI)	*P*-value
Prehypertension								
AIP*10	1.17 (1.15, 1.19)	<0.001	1.10 (1.08, 1.12)	<0.001	1.11 (1.09, 1.13)	<0.001	1.05 (1.03, 1.07)	<0.001
Q1	Ref		Ref		Ref		Ref	
Q2	1.55 (1.36, 1.77)	<0.001	1.31 (1.14, 1.50)	<0.001	1.32 (1.15, 1.52)	0.001	1.17 (1.02, 1.35)	0.029
Q3	2.22 (1.93, 2.55)	<0.001	1.62 (1.40, 1.88)	<0.001	1.66 (1.43, 1.93)	<0.001	1.30 (1.11, 1.52)	0.001
Q4	3.61 (3.09, 4.21)	<0.001	2.34 (1.99, 2.75)	<0.001	2.47 (2.09, 2.93)	<0.001	1.58 (1.32, 1.90)	<0.001
P for trend	5.74 (4.72, 6.99)	<0.001	3.07 (2.49, 3.77)	<0.001	3.30 (2.67, 4.09)	<0.001	1.82 (1.44, 2.30)	<0.001
Hypertension								
AIP*10	1.30 (1.27, 1.32)	<0.001	1.23 (1.21, 1.26)	<0.001	1.24 (1.22, 1.27)	<0.001	1.11 (1.09, 1.14)	<0.001
Q1	Ref		Ref		Ref		Ref	
Q2	2.14 (1.85, 2.49)	<0.001	1.63 (1.38, 1.94)	<0.001	1.68 (1.41, 2.00)	<0.001	1.25 (1.04, 1.50)	0.019
Q3	4.08 (3.51, 4.74)	<0.001	2.63 (2.21, 3.12)	<0.001	2.72 (2.27, 3.25)	<0.001	1.56 (1.29, 1.89)	<0.001
Q4	8.70 (7.41, 10.22)	<0.001	5.68 (4.72, 6.82)	<0.001	6.01 (4.96, 7.26)	<0.001	2.30 (1.86, 2.84)	<0.001
P for trend	18.33 (14.97, 22.43)	<0.001	10.36 (8.22, 13.07)	<0.001	11.1 (8.72, 14.12)	<0.001	3.08 (2.36, 4.04)	<0.001

Model 1 adjusts for age groups and sex. Model 2 adjusts for Model 1 + residential location, education levels, smoking, alcohol consumption, marital status, per capita annual household income, physical activity, and sleep status. Model 3 adjusts for Model 2 + BMI groups, WC groups, TC, Cr, UA, and HbA1c groups. AIP, atherogenic index of plasma; OR, odds ratio; CI, confidence interval; BMI, body mass index; WC, waist circumference; TC, total cholesterol; Cr, creatinine; UA, uric acid; HbA1c, hemoglobin A1c.

### Subgroup analyses

**Figure 2:**
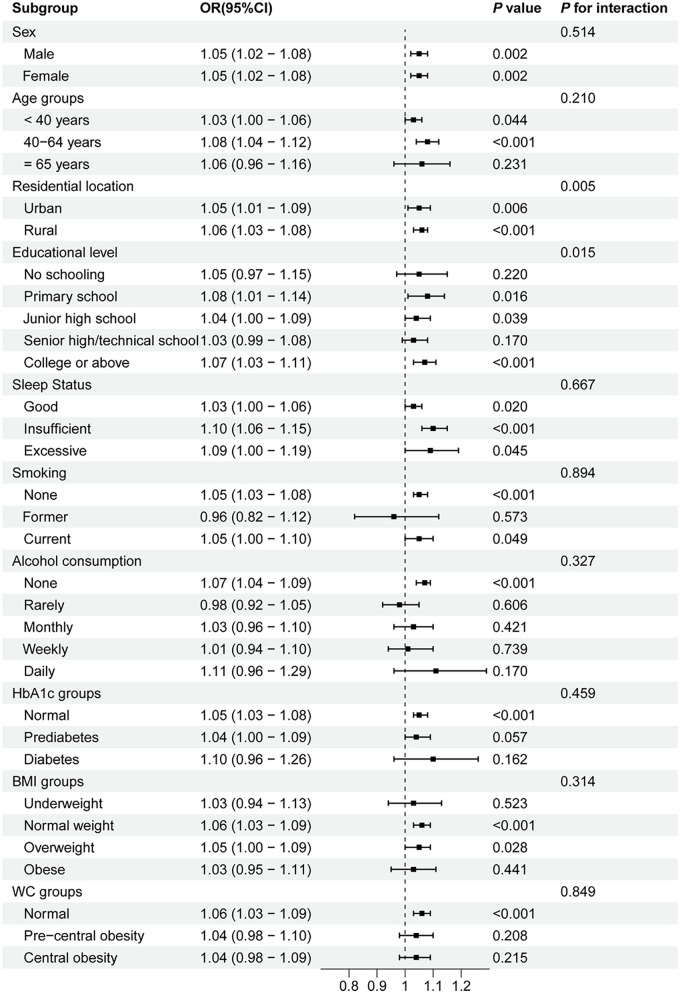
Subgroup analyses of the association between the AIP and prehypertension. AIP, atherogenic index of plasma; OR, odds ratio; CI, confidence interval; WC, waist circumference; BMI, body mass index; HbA1c, hemoglobin A1c.

**Figure 3:**
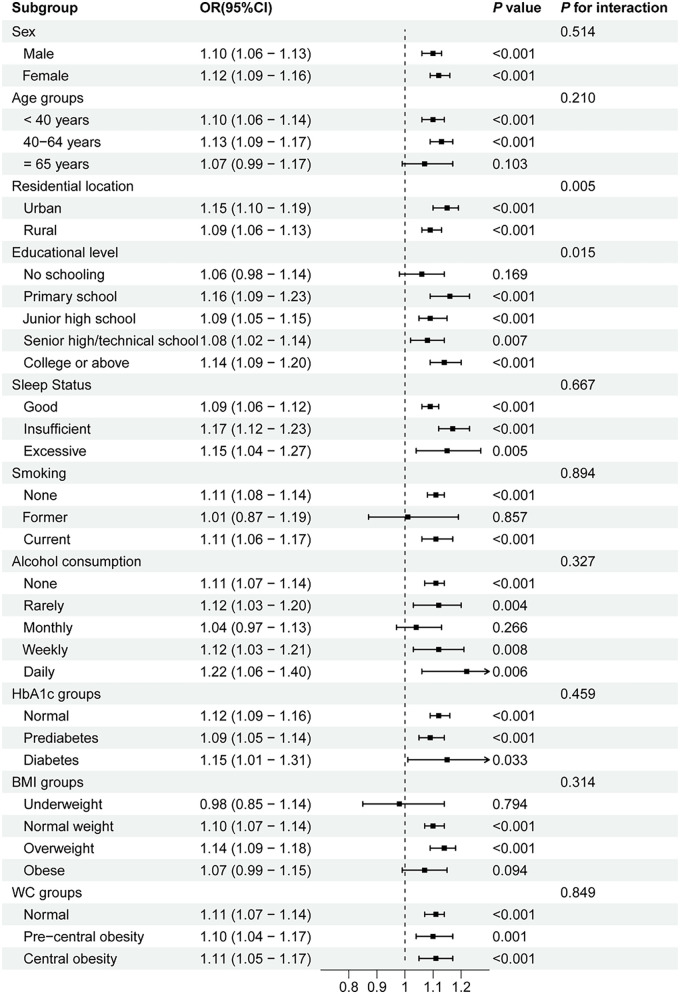
Subgroup analyses of the association between the AIP and hypertension. AIP, atherogenic index of plasma; OR, odds ratio; CI, confidence interval; WC, waist circumference; BMI, body mass index; HbA1c, hemoglobin A1c.

Subgroup analyses and interaction tests were conducted based on age, sex, residential location, educational level, sleep status, smoking, alcohol consumption, HbA1c groups, BMI groups, and WC groups to assess whether the relationship between the AIP and prehypertension/hypertension was consistent across different population subgroups. As shown in Figure 2, the positive association between the AIP and prehypertension remained stable in all subgroups, except among former smokers and individuals who rarely consumed alcohol. A stratified analysis demonstrated stronger associations between the AIP and prehypertension in the 40–64-year age group (OR = 1.08, *P* < 0.001), individuals with insufficient sleep status (OR = 1.10, *P* < 0.001), non-smokers (OR = 1.05, *P* < 0.001), non-alcohol drinkers (OR = 1.07, *P* < 0.001), and those with normal HbA1c levels (OR = 1.05, *P* < 0.001), normal BMI (OR = 1.06, *P* < 0.001), and normal WC (OR = 1.06, *P* < 0.001). Moreover, robust associations between the AIP and hypertension were consistently observed among middle-aged adults (40–64 years, OR = 1.13, *P* < 0.001), participants with primary school education (OR = 1.16, *P* < 0.001), current smokers (OR = 1.11, *P* < 0.001), daily alcohol consumers (OR = 1.22, *P* = 0.006), individuals with HbA1c < 5.7% (OR = 1.15, *P* < 0.001), those with insufficient sleep (OR = 1.17, *P* = 0.001), overweight participants (OR = 1.14, *P* < 0.001), and all WC categories (OR = 1.10–1.11, all *P* < 0.001) (shown in Figure 3). Significant interaction effects were observed between the AIP and residential location (*P* for interaction = 0.005), as well as educational level (*P* for interaction = 0.015), on blood pressure classification. Quartile-based analyses further confirmed a graded increase in the risk of prehypertension and hypertension across ascending AIP categories, supporting the robustness of the main findings (Supplementary Tables S1, S2).

### RCS analyses

**Figure 4:**
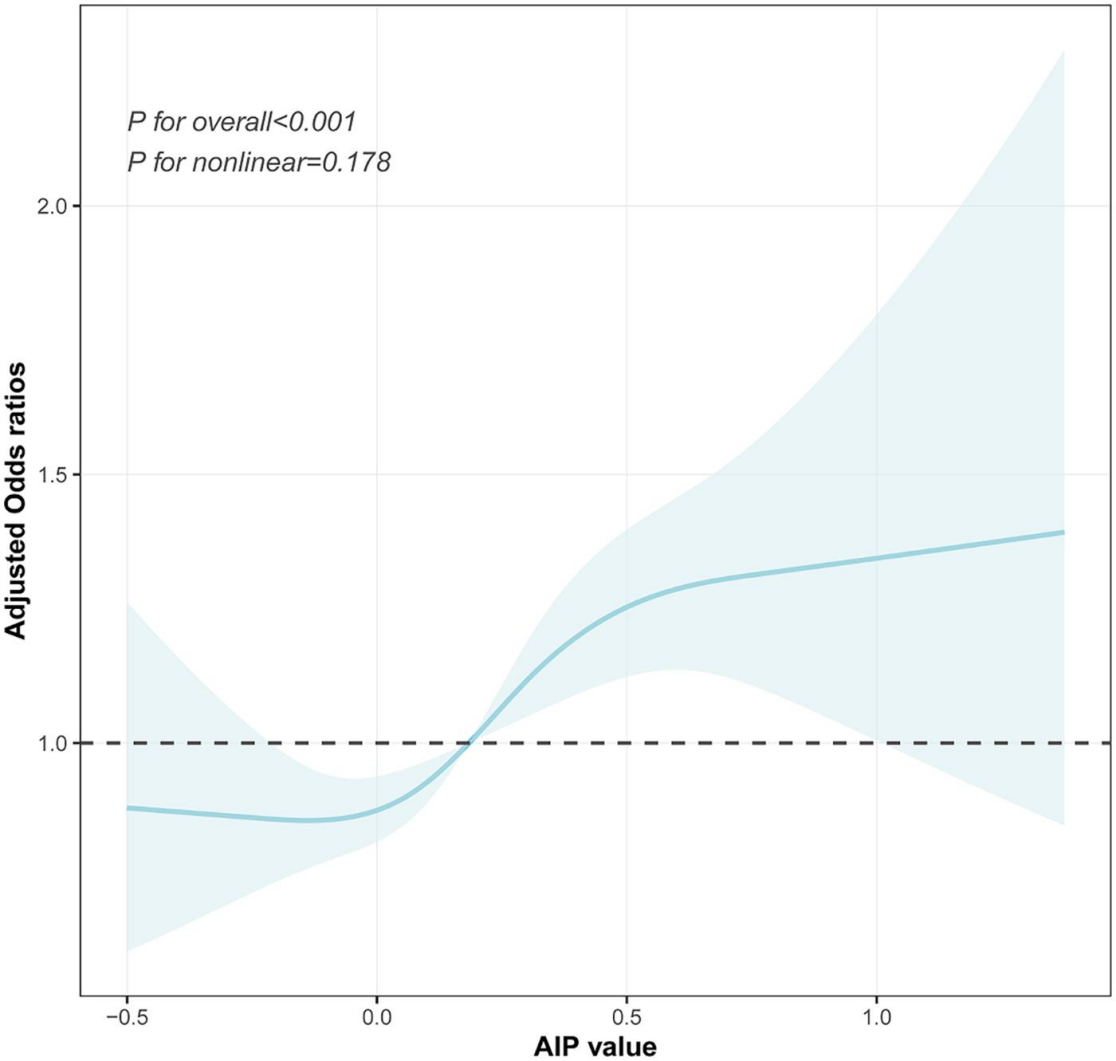
Visualizing the relationship between the AIP and prehypertension using a four-knot RCS (adjusted for Model 3). AIP, atherogenic index of plasma.

**Figure 5:**
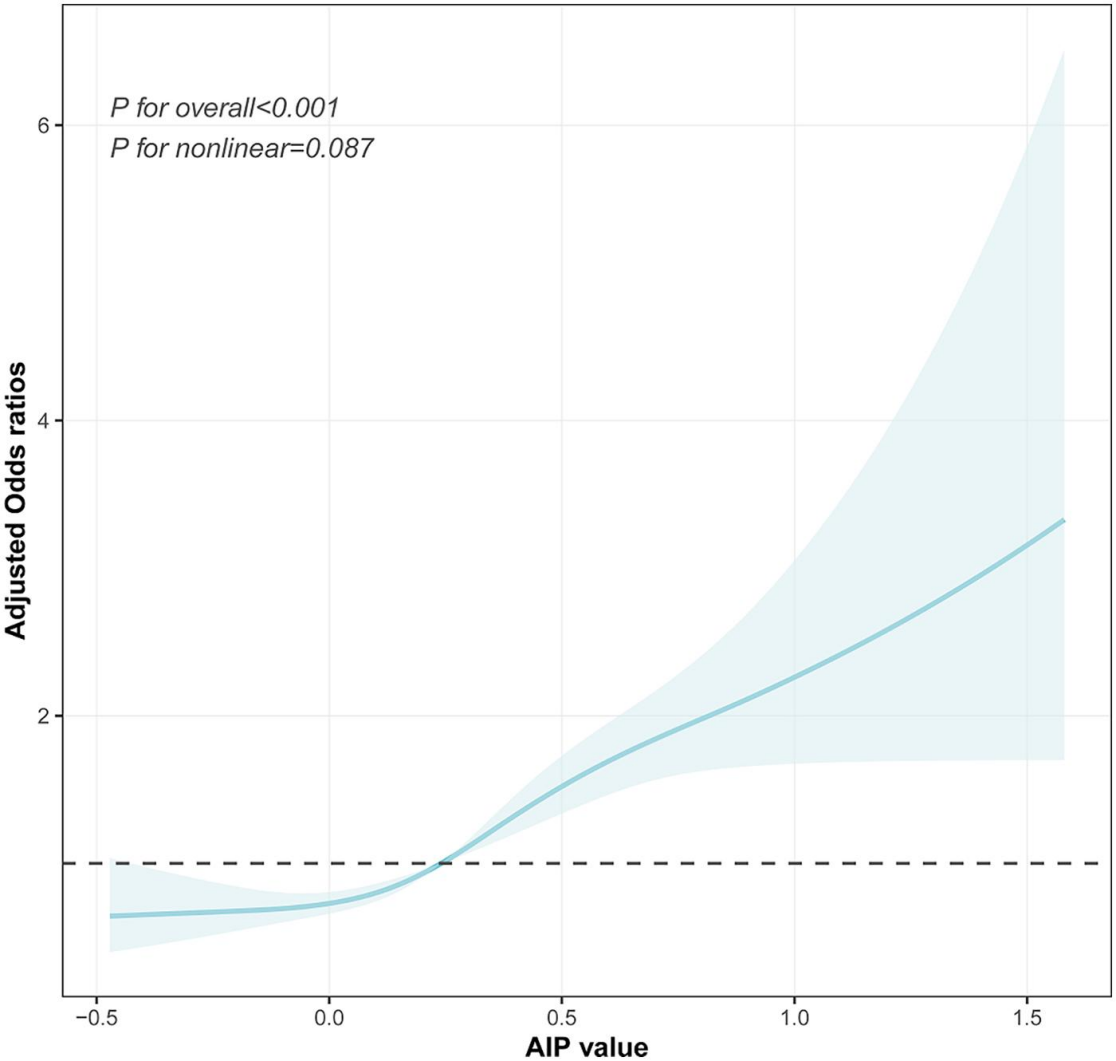
Visualizing the relationship between the AIP and hypertension using a four-knot RCS (adjusted for Model 3). AIP, atherogenic index of plasma.

The dose–response relationship between the AIP and prehypertension or hypertension was further explored using a four-knot RCS model. As shown in Figure 4, in the fully adjusted Model 3, the risk of prehypertension increased with an increasing AIP (*P* for total <0.001), and no non-linear association was observed (*P* for non-linea*r* = 0.178). Similarly, after the full adjustment, the AIP was positively associated with the risk of hypertension in a dose–response manner (*P* for total <0.001, shown in Figure 5), with no evidence of a non-linear relationship (*P* for non-linear = 0.087, shown in Figure 5).

### ROC curve analyses

**Figure 6:**
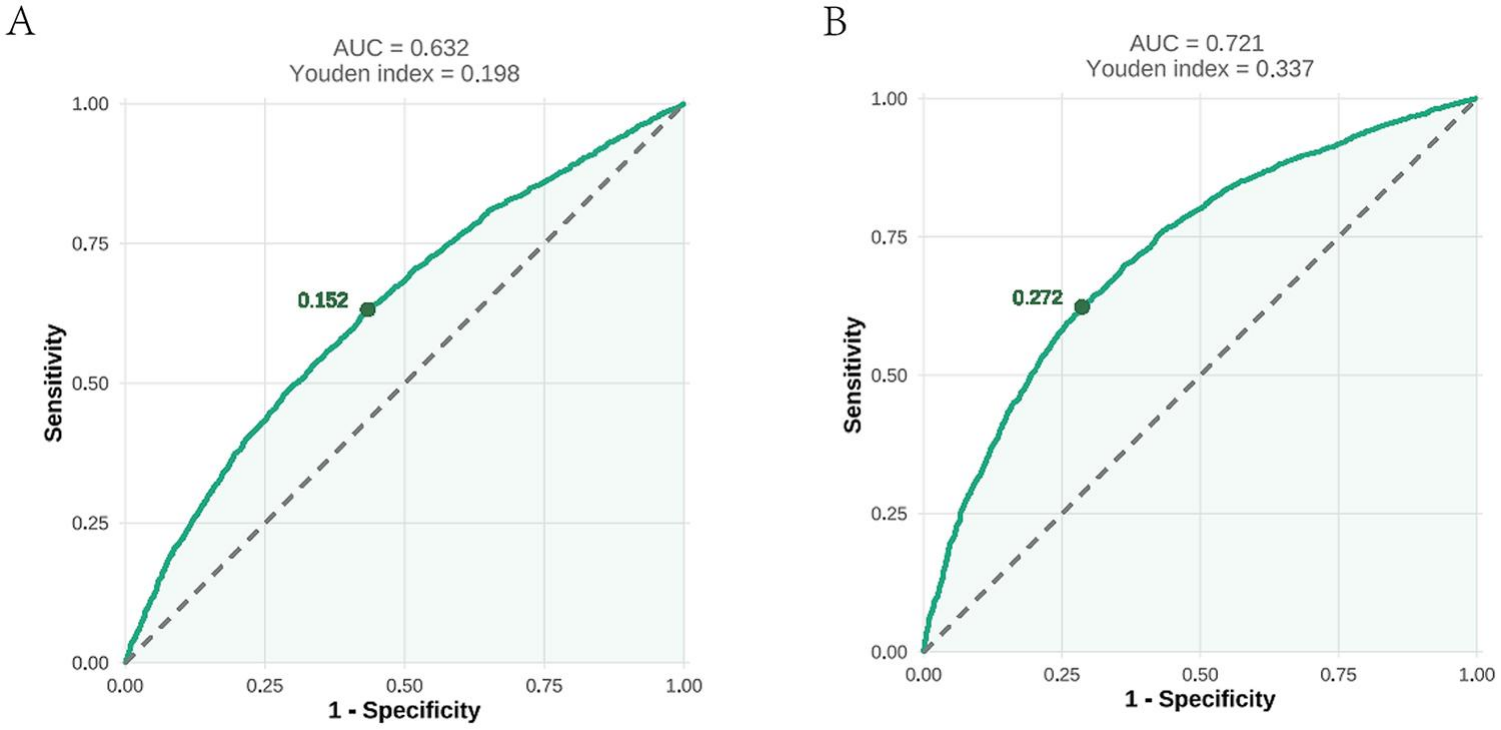
An ROC analysis of the AIP for predicting prehypertension **(A)** and hypertension **(B)**. ROC, receiver operating characteristic; AUC, area under the curve; AIP, atherogenic index of plasma.

As illustrated in Figure 6A, the ROC curve analysis for the AIP in predicting prehypertension showed an AUC of 0.632 (95% CI: 0.618–0.646; *P* < 0.001) with an optimal cutoff value of 0.152 (sensitivity=0.632; specificity = 0.566). These results indicated the limited diagnostic utility of the AIP for prehypertension assessment. For hypertension (Figure 6B), the ROC analysis demonstrated improved discriminatory performance, with an AUC of 0.721 (95% CI: 0.708–0.734; *P* < 0.001) at a cutoff value of 0.272 (sensitivity = 0.623; specificity = 0.714).

### Mediation analyses through WBCs and NE

**Figure 7:**
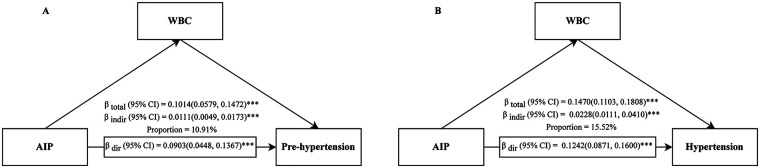
Mediation analysis of the association between the AIP and prehypertension **(A)**/hypertension **(B)** through WBCs. AIP, atherogenic index of plasma; CI, confidence interval; WBC, white blood cell; ***P < 0.001.

Mediation analyses revealed that WBCs played a significant mediating role in the relationship between the AIP and blood pressure status. For prehypertension, WBCs mediated 11.87% (95% CI: 5.40%–25.89%) of the total effect; this proportion increased to 15.32% (95% CI: 7.32%–27.96%) for hypertension (Figure 7 and Supplementary Table S3). In contrast, the mediating effect of NE was not significant in either model (*P* > 0.05) (Supplementary Table S3).

### Sensitivity analyses

For hypertension, the four-knot model showed a significantly better fit than the three-knot model (likelihood ratio test *P* = 0.030), with the lowest AIC among candidate models (Table 3). For prehypertension, differences in the model fit between the three- and four-knot models were modest, and no strong evidence of non-linearity was observed (Table 3). Increasing the number of knots to five did not further improve model performance for either outcome. Overall, a four-knot restricted cubic spline model was selected as a balance between the model fit and parsimony. Sensitivity analyses suggested that the estimated mediation effect of WBCs for prehypertension was sensitive to potential unmeasured confounding (*ρ* = 0.1), indicating that this finding should be interpreted with caution. In contrast, the mediation effect for hypertension demonstrated moderate robustness to unmeasured confounding (*ρ* = 0.3) (Supplementary Table S4).

**Table 3:** Model performance and statistical comparisons of RCS models with varying knot specifications.

Knots	AIC	BIC	*P* for non-linear	Likelihood ratio test (vs. previous model)
Prehypertension				
3	7,559.22	7,821.93	0.961	Ref
4	7,557.79	7,827.24	0.178	*χ*^2^ = 3.43, *P* = 0.064
5	7,559.38	7,835.57	0.276	χ^2^ = 0.41, *P* = 0. 523
Hypertension				
3	4,968.22	5,230.78	0.763	Ref
4	4,965.53	5,234.82	0.089	χ^2^ = 4.69, *P* = 0.030
5	4,967.24	5,243.27	0.161	χ^2^ = 0.29, *P* = 0.594

RCS, restricted cubic splines; AIC, Akaike information criterion; BIC, Bayesian information criterion.

## Discussion

In this population-based cross-sectional study, our results indicated positive and linear associations between the AIP and the risk of prehypertension and hypertension among the Chinese population aged 18 years and above. Furthermore, subgroup analyses revealed that the association between the AIP and the risk of prehypertension and hypertension was particularly pronounced in individuals with a healthy glucose metabolism and normal body size and in specific behavioral subgroups, and mediation analyses suggested that this association may be partly explained by systemic inflammation, as reflected by WBCs.

The AIP reflects the core features of atherogenic dyslipidemia and is regarded as a novel biomarker for the early diagnosis of CVD events (47). Existing evidence indicates that the AIP is closely associated with coronary artery disease, type 2 diabetes, non-alcoholic fatty liver disease, and other conditions (26, 27, 48). From a clinical perspective, the AIP serves as a useful and integrative marker that captures the balance between atherogenic and antiatherogenic lipid fractions, thereby facilitating early risk stratification for cardiovascular diseases, including hypertension (49). From a pathophysiological standpoint, however, an elevated AIP reflects an unfavorable lipid milieu characterized by increased triglyceride-rich lipoproteins and reduced HDL-C, which may contribute to endothelial dysfunction, arterial stiffness, and low-grade systemic inflammation, all of which are key mechanisms involved in the development and progression of hypertension (50–52). In line with these mechanisms, our results demonstrated a significant positive association of the AIP with hypertension/prehypertension, consistent with, and further extend, the results from multiple previous population-based studies (28–39). Furthermore, we did not observe any evidence of a non-linear association between the AIP and elevated blood pressure.

Subgroup analyses revealed consistent AIP-blood pressure associations across most subgroups, indicating pervasive stability. Notably, a stronger association occurred in individuals aged 40–64, insufficient sleep, normal HbA1c, normal BMI/overweight status, and normal WC. Moreover, the association exhibited significant age dependency, being significant only in groups under 65 years (strongest at 40–64), which aligns with East Asian studies (29, 39). This phenomenon suggests that middle age may represent a critical window period where AIP-related metabolic dysregulation drives blood pressure elevation, as the active insulin resistance and vascular endothelial dysfunction characteristic of this stage synergize with the atherogenic lipid profile to cause damage (53). The absence of an association in the elderly group may be attributed not only to age-related shifts in pathological mechanisms but also to polypharmacy and survival bias (54, 55). The attenuated association in obesity is consistent with what is reported in Cheng et al (32). Such a pattern may reflect the prevalent “metabolically obese normal weight” phenotype (56), wherein many normal-BMI individuals have undetected visceral adiposity or lipid disorders, increasing susceptibility to AIP effects. The stronger association observed in individuals with normal HbA1c and normal WC suggests that within populations characterized by normal body size and healthy glucose metabolism, the AIP may more sensitively reflect the comorbid features of lipid metabolism disorders and blood pressure abnormalities. In addition, the strong association between the AIP and hypertension observed among current smokers and daily alcohol consumers suggests that the synergistic effect of the AIP and behavioral factors may accelerate the progression of hypertension. We observed significant interaction effects between the AIP and both residential location and educational level on blood pressure classification. Interestingly, the association between the AIP and elevated blood pressure was stronger among participants with higher educational levels and those living in urban areas. This may reflect lifestyle patterns such as higher-fat diets, physical inactivity, and greater occupational stress, which could amplify the impact of atherogenic dyslipidemia on blood pressure (57, 58). It is noteworthy that gender did not exert a significant modifying effect on the association between AIP and blood pressure in the present study, while the Japanese study showed that this association was stronger in females (39). The reason for this may be that the population heterogeneity in genetic background, environmental exposures (such as dietary patterns), or metabolic status may render this pathological mechanism universally applicable across genders (59, 60).

In our study, a mediation analysis demonstrated that WBCs significantly mediated the association between the AIP and both pre-hypertension and hypertension, indicating that systemic inflammation partially underlies the effect of the AIP on blood pressure regulation. This association may be mediated through mechanisms involving lipid metabolism disorders combined with vascular damage and inflammatory pathways. Specifically, a high TG/HDL-C ratio promotes oxidative stress and endothelial dysfunction, subsequently driving vascular remodeling and increased blood pressure (61). As for inflammatory pathways, they may act as a mediating mechanism linking the AIP to elevated blood pressure rather than simply a parallel factor (62,63). Studies from Turkey also suggested that the AIP was closely linked to inflammatory markers like C-reactive protein (28).

Our study was strengthened by its large sample size, rigorous multivariate adjustments, mediation analyses, and sensitivity analyses, which collectively enhanced the reliability of the results. However, several limitations should be acknowledged in this study: the cross-sectional design precluded causal inference, necessitating validation of the temporal relationship between the AIP and blood pressure progression through prospective cohorts; the lack of dietary data (e.g., salt intake) may obscure pathways linking the AIP to hypertension; and the findings were specific to adults in Fujian Province, China, with potential under-representation of extreme metabolic phenotypes or subgroups such as individuals with severe obesity. Although WBCs and NE were used to assess the mediating role of inflammation, a more comprehensive measurement of inflammatory factors could provide a deeper understanding of the role of inflammation in the mediation process. At the same time, future studies should employ longitudinal designs to clarify causality and integrate metabolomic approaches to unravel molecular mechanisms. Targeted interventions for high-risk subgroups (e.g., urban populations and those with high educational backgrounds) combining lipid management and lifestyle modifications may yield enhanced cost-effectiveness within health economics frameworks. Furthermore, multimarker models incorporating the AIP and insulin resistance indices may refine hypertension risk prediction models.

## Conclusion

The AIP was positively associated with prehypertension and hypertension, and this association was stronger in the 40–64 age group, in individuals with a healthy glucose metabolism and normal body size, and in specific behavioral subgroups. White blood cells mediated this association. Although the cross-sectional design constrained causal inference, the AIP, as a biomarker of lipid metabolism dysregulation, may serve as a reference for early hypertension detection.

## Data Availability

The raw data supporting the conclusions of this article will be made available by the authors, without undue reservation.
