# Supplementary material for: Association between the atherogenic index of plasma and prehypertension or hypertension among adults in Fujian, China: a population-based cross-sectional study
Source: Front Cardiovasc Med. 2026 Feb 5;13:1708190. doi: 10.3389/fcvm.2026.1708190 (PMC12916675; doi:10.3389/fcvm.2026.1708190)
Supplement: Supplementary file 1 [file Table1.docx]

| **Table S1 Subgroup analysis of the associations between AIP and Prehypertension.** | | | | | | | | |
| --- | --- | --- | --- | --- | --- | --- | --- | --- |
| Subgroup | Count | OR(95%CI) | | | | P value (Q4 vs Q1) | P for trend | *P* for interaction |
|  |  | Q1 | Q2 | Q3 | Q4 |  |  |  |
| Sex |  |  |  |  |  |  |  | 0.214 |
| Male | 2836 | Ref | 1.15 (0.91-1.46) | 1.26 (0.99-1.60) | 1.52 (1.16-1.99) | 0.003 | 0.003 |  |
| Female | 3389 | Ref | 1.16 (0.96-1.39) | 1.26 (1.02-1.56) | 1.60 (1.22-2.09) | <0.001 | <0.001 |  |
| Age Groups |  |  |  |  |  |  |  | 0.176 |
| < 40 | 3689 | Ref | 1.11 (0.92-1.34) | 1.16 (0.94-1.42) | 1.48 (1.15-1.91) | 0.002 | 0.008 |  |
| ≥ 40 and <65 | 2107 | Ref | 1.22 (0.94-1.57) | 1.49 (1.14-1.95) | 1.70 (1.27-2.29) | <0.001 | <0.001 |  |
| ≥ 65 | 429 | Ref | 1.32 (0.70-2.47) | 1.44 (0.74-2.77) | 1.57 (0.73-3.38) | 0.243 | 0.208 |  |
| Residential Location |  |  |  |  |  |  |  | 0.141 |
| Urban | 2396 | Ref | 1.29 (1.01-1.65) | 1.39 (1.07-1.82) | 1.56 (1.16-2.10) | 0.003 | 0.003 |  |
| Rural | 3829 | Ref | 1.11 (0.93-1.33) | 1.25 (1.03-1.51) | 1.64 (1.29-2.08) | <0.001 | <0.001 |  |
| Educational level |  |  |  |  |  |  |  | 0.024 |
| No schooling | 426 | Ref | 1.15 (0.65-2.06) | 1.54 (0.82-2.89) | 1.09 (0.55-2.16) | 0.800 | 0.505 |  |
| Primary school | 785 | Ref | 1.01 (0.67-1.52) | 1.48 (0.95-2.31) | 1.54 (0.92-2.58) | 0.098 | 0.036 |  |
| Junior high school | 1441 | Ref | 1.44 (1.06-1.96) | 1.14 (0.83-1.58) | 1.69 (1.17-2.44) | 0.005 | 0.027 |  |
| Senior high / Technical school | 1306 | Ref | 1.39 (1.01-1.92) | 1.52 (1.08-2.15) | 1.33 (0.88-2.00) | 0.172 | 0.083 |  |
| College or above | 2267 | Ref | 0.99 (0.78-1.26) | 1.19 (0.90-1.56) | 2.02 (1.45-2.83) | <0.001 | <0.001 |  |
| Sleep Status |  |  |  |  |  |  |  | 0.434 |
| Good | 4282 | Ref | 1.13 (0.95-1.34) | 1.14 (0.94-1.37) | 1.34 (1.07-1.67) | 0.010 | 0.016 |  |
| Insufficient | 1500 | Ref | 1.45 (1.07-1.95) | 1.73 (1.25-2.38) | 2.30 (1.58-3.35) | <0.001 | <0.001 |  |
| Excessive | 443 | Ref | 0.82 (0.46-1.47) | 1.84 (0.96-3.54) | 1.98 (0.91-4.34) | 0.087 | 0.025 |  |
| Smoking |  |  |  |  |  |  |  | 0.028 |
| None | 4809 | Ref | 1.19 (1.02-1.39) | 1.37 (1.15-1.64) | 1.58 (1.28-1.96) | <0.001 | <0.001 |  |
| Former | 164 | Ref | 1.46 (0.44-4.85) | 1.80 (0.53-6.10) | 1.52 (0.43-5.40) | 0.521 | 0.472 |  |
| Current | 1252 | Ref | 1.02 (0.69-1.51) | 0.96 (0.66-1.40) | 1.33 (0.87-2.02) | 0.190 | 0.263 |  |
| Alcohol Consumption |  |  |  |  |  |  |  | 0.383 |
| None | 4284 | Ref | 1.28 (1.08-1.52) | 1.44 (1.19-1.73) | 1.83 (1.46-2.29) | <0.001 | <0.001 |  |
| Rarely | 807 | Ref | 0.96 (0.64-1.45) | 0.77 (0.49-1.22) | 0.81 (0.46-1.41) | 0.450 | 0.267 |  |
| Monthly | 601 | Ref | 0.67 (0.39-1.13) | 1.00 (0.58-1.70) | 1.24 (0.68-2.27) | 0.486 | 0.269 |  |
| Weekly | 413 | Ref | 0.95 (0.48-1.89) | 0.85 (0.43-1.72) | 1.12 (0.54-2.32) | 0.761 | 0.831 |  |
| Daily | 120 | Ref | 1.30 (0.37-4.60) | 5.31 (1.40-20.08) | 1.62 (0.43-6.12) | 0.474 | 0.233 |  |
| HbA1c Groups |  |  |  |  |  |  |  | 0.232 |
| Normal | 4689 | Ref | 1.15 (0.98-1.35) | 1.26 (1.06-1.51) | 1.65 (1.32-2.05) | <0.001 | <0.001 |  |
| Prediabetes | 1370 | Ref | 1.38 (0.97-1.97) | 1.39 (0.97-1.98) | 1.56 (1.06-2.30) | 0.025 | 0.043 |  |
| Diabetes | 166 | Ref | 0.75 (0.21-2.66) | 2.40 (0.62-9.27) | 2.01 (0.55-7.30) | 0.291 | 0.097 |  |
| BMI Groups |  |  |  |  |  |  |  | 0.594 |
| Underweight | 491 | Ref | 0.99 (0.61-1.62) | 1.58 (0.84-3.00) | 2.67 (0.78-9.09) | 0.116 | 0.092 |  |
| Normal weight | 3559 | Ref | 1.16 (0.97-1.38) | 1.28 (1.05-1.56) | 1.63 (1.27-2.09) | <0.001 | <0.001 |  |
| Overweight | 1665 | Ref | 1.39 (1.00-1.93) | 1.46 (1.06-2.03) | 1.61 (1.13-2.30) | 0.008 | 0.014 |  |
| Obese | 510 | Ref | 1.20 (0.57-2.52) | 1.14 (0.56-2.34) | 1.7 (0.83-3.50) | 0.148 | 0.119 |  |
| WC Groups |  |  |  |  |  |  |  | 0.755 |
| Normal | 4369 | Ref | 1.14 (0.98-1.34) | 1.31 (1.09-1.57) | 1.65 (1.32-2.08) | <0.001 | <0.001 |  |
| Pre-central obesity | 921 | Ref | 1.30 (0.82-2.06) | 1.21 (0.77-1.91) | 1.49 (0.90-2.48) | 0.120 | 0.241 |  |
| Central obesity | 935 | Ref | 1.44 (0.85-2.43) | 1.58 (0.95-2.64) | 1.76 (1.05-2.96) | 0.033 | 0.044 |  |
| AIP, Atherogenic Index of plasma; OR, odds ratio; CI, confidence interval; WC, Waist Circumference; BMI, Body Mass Index; HbA1c, Hemoglobin A1c. | | | | | | | | |

| **Table S2 Subgroup analysis of the associations between AIP and Hypertension.** | | | | | | | | |
| --- | --- | --- | --- | --- | --- | --- | --- | --- |
| Subgroup | Count | OR(95%CI) | | | | *P* value (Q4 vs Q1) | *P* for trend | *P* for interaction |
|  |  | Q1 | Q2 | Q3 | Q4 |  |  |  |
| Gender |  |  |  |  |  |  |  | 0.214 |
| Male | 2795 | Ref | 1.08 (0.81-1.43) | 1.16 (0.88-1.54) | 1.87 (1.38-2.52) | <0.001 | <0.001 |  |
| Female | 3405 | Ref | 1.36 (1.05-1.74) | 1.94 (1.49-2.54) | 2.58 (1.88-3.52) | <0.001 | <0.001 |  |
| Age Groups |  |  |  |  |  |  |  | 0.176 |
| < 40 | 2629 | Ref | 0.95 (0.69-1.32) | 1.01 (0.72-1.42) | 1.95 (1.36-2.80) | <0.001 | <0.001 |  |
| ≥ 40 and < 65 | 2345 | Ref | 1.45 (1.09-1.91) | 2.05 (1.53-2.73) | 2.62 (1.92-3.57) | <0.001 | <0.001 |  |
| ≥ 65 | 1226 | Ref | 1.42 (0.80-2.53) | 1.70 (0.93-3.11) | 1.79 (0.88-3.64) | 0.105 | 0.074 |  |
| Residential Location |  |  |  |  |  |  |  | 0.141 |
| Urban | 2314 | Ref | 1.44 (1.02-2.02) | 2.00 (1.42-2.83) | 2.88 (2.00-4.15) | <0.001 | <0.001 |  |
| Rural | 3886 | Ref | 1.21 (0.96-1.51) | 1.42 (1.12-1.79) | 2.12 (1.62-2.78) | <0.001 | <0.001 |  |
| Educational level |  |  |  |  |  |  |  | 0.024 |
| No schooling | 881 | Ref | 1.29 (0.74-2.24) | 1.61 (0.88-2.95) | 1.26 (0.67-2.39) | 0.475 | 0.319 |  |
| Primary school | 1129 | Ref | 1.16 (0.76-1.76) | 2.07 (1.32-3.25) | 2.46 (1.47-4.09) | <0.001 | <0.001 |  |
| Junior high school | 1427 | Ref | 1.55 (1.05-2.28) | 1.55 (1.05-2.29) | 2.52 (1.66-3.82) | <0.001 | <0.001 |  |
| Senior high / Technical school | 1074 | Ref | 1.20 (0.76-1.90) | 1.41 (0.89-2.25) | 1.63 (0.98-2.70) | 0.059 | 0.016 |  |
| College or above | 1689 | Ref | 0.98 (0.63-1.53) | 1.35 (0.86-2.11) | 2.97 (1.84-4.81) | <0.001 | <0.001 |  |
| Sleep Status |  |  |  |  |  |  |  | 0.434 |
| Good | 4023 | Ref | 1.19 (0.94-1.50) | 1.45 (1.14-1.84) | 1.94 (1.49-2.53) | <0.001 | <0.001 |  |
| Insufficient | 1735 | Ref | 1.42 (1.01-2.01) | 1.87 (1.31-2.67) | 3.34 (2.23-4.98) | <0.001 | <0.001 |  |
| Excessive | 442 | Ref | 1.29 (0.62-2.68) | 1.89 (0.85-4.22) | 3.72 (1.50-9.23) | 0.005 | 0.004 |  |
| Smoking |  |  |  |  |  |  |  | 0.028 |
| None | 4629 | Ref | 1.19 (0.96-1.46) | 1.63 (1.31-2.04) | 2.25 (1.76-2.89) | <0.001 | <0.001 |  |
| Former | 224 | Ref | 4.63 (1.27-16.90) | 3.83 (1.04-14.16) | 1.93 (0.50-7.47) | 0.342 | 0.631 |  |
| Current | 1347 | Ref | 1.11 (0.71-1.72) | 1.18 (0.77-1.81) | 2.19 (1.38-3.47) | <0.001 | <0.001 |  |
| Alcohol Consumption |  |  |  |  |  |  |  | 0.383 |
| None | 4271 | Ref | 1.33 (1.07-1.66) | 1.66 (1.31-2.08) | 2.22 (1.71-2.88) | <0.001 | <0.001 |  |
| Rarely | 653 | Ref | 1.07 (0.59-1.95) | 1.11 (0.60-2.08) | 2.18 (1.09-4.33) | 0.027 | 0.02 |  |
| Monthly | 518 | Ref | 0.84 (0.42-1.67) | 1.34 (0.69-2.62) | 1.71 (0.82-3.54) | 0.151 | 0.077 |  |
| Weekly | 513 | Ref | 1.08 (0.50-2.33) | 1.33 (0.62-2.85) | 2.51 (1.15-5.47) | 0.021 | 0.008 |  |
| Daily | 245 | Ref | 1.39 (0.45-4.27) | 3.29 (1.00-10.79) | 2.99 (0.90-9.98) | 0.074 | 0.03 |  |
| HbA1c Groups |  |  |  |  |  |  |  | 0.232 |
| Normal | 3868 | Ref | 1.13 (0.90-1.42) | 1.52 (1.20-1.93) | 2.52 (1.93-3.28) | <0.001 | <0.001 |  |
| Prediabetes | 1844 | Ref | 1.51 (1.05-2.17) | 1.51 (1.05-2.17) | 2.12 (1.43-3.13) | <0.001 | <0.001 |  |
| Diabetes | 488 | Ref | 1.11 (0.33-3.72) | 4.31 (1.15-16.11) | 3.02 (0.86-10.61) | 0.085 | 0.031 |  |
| BMI Groups |  |  |  |  |  |  |  | 0.594 |
| Normal weight | 3165 | Ref | 1.15 (0.91-1.46) | 1.45 (1.13-1.86) | 2.04 (1.52-2.74) | <0.001 | <0.001 |  |
| Underweight | 397 | Ref | 0.5 (0.20-1.26) | 1.62 (0.53-4.92) | 2.74 (0.55-13.68) | 0.218 | 0.346 |  |
| Overweight | 1883 | Ref | 1.52 (1.04-2.23) | 1.82 (1.25-2.64) | 2.95 (1.99-4.37) | <0.001 | <0.001 |  |
| Obese | 755 | Ref | 2.05 (0.93-4.55) | 2.01 (0.94-4.32) | 2.64 (1.23-5.67) | 0.013 | 0.045 |  |
| WC Groups |  |  |  |  |  |  |  | 0.755 |
| Normal | 4369 | Ref | 1.21 (0.97-1.50) | 1.59 (1.26-2.01) | 2.15 (1.64-2.83) | <0.001 | <0.001 |  |
| Pre-central obesity | 921 | Ref | 1.20 (0.71-2.01) | 1.12 (0.67-1.88) | 2.27 (1.31-3.96) | 0.004 | 0.002 |  |
| Central obesity | 935 | Ref | 1.78 (1.04-3.04) | 2.27 (1.35-3.82) | 2.96 (1.74-5.01) | <0.001 | <0.001 |  |
| AIP, Atherogenic Index of plasma; OR, odds ratio; CI, confidence interval; WC, Waist Circumference; BMI, Body Mass Index; HbA1c, Hemoglobin A1c. | | | | | | | | |

| **Table S3 The mediating effects of inflammatory factors on the association between AIP and pre-hypertension/hypertension.** | | | | | | | | |
| --- | --- | --- | --- | --- | --- | --- | --- | --- |
| **Inflammatory indicators** | **ACME (95% CI)** | **P value** | **ADE  (95% CI)** | **P value** | **Total Effect  (95% CI)** | **P value** | **Mediated proportion (95% CI)** | ***P* value** |
| **Pre-hypertension** |  |  |  |  |  |  |  |  |
| WBC | 0.0111 (0.00489, 0.0173) | <0.001 | 0.0903 (0.0448, 0.1367) | <0.001 | 0.1014 (0.0579, 0.1472) | <0.001 | 10.91%  (4.73%, 23.32%) | <0.001 |
| NE | 0.000012 (-0.00025, 0.0117) | 0.638 | 0.1014 (0.0542, 0.1455) | <0.001 | 0.1014 (0.0577, 0.1470) | <0.001 | 0.00012%  (-0.27%, 13.36%) | 0.638 |
| **Hypertension** |  |  |  |  |  |  |  |  |
| WBC | 0.0228 (0.0111, 0.0410) | <0.001 | 0.1242  (0.0871, 0.1600) | <0.001 | 0.1470 (0.1103, 0.1808) | <0.001 | 15.52% (7.30%, 29.27%) | <0.001 |
| NE | 0.00041 (-0.00004, 0.0161) | 0.166 | 0.1429 (0.1015, 0.1764) | <0.001 | 0.1433 (0.1061, 0.1801) | <0.001 | 0.29% (-0.03%, 12.06%) | 0.166 |
| All covariates including age groups, sex, residential location, education levels, smoking, alcohol consumption, sleep status, marital status, per capita annual household income, physical activity, BMI groups, WC groups, TC, Cr, UA and HbA1c groups were adjusted in this model.  CI, confidence interval; WBC, white blood cell; NE, Neutrophils; BMI, body mass index; WC, waist circumference; TC, total cholesterol; Cr, creatinine; HbA1c, hemoglobin A1c. | | | | | | | | |

**Table S4 Sensitivity Analysis Results of WBC Mediation Effects**

| **Outcome** | **Sensitivity Threshold (ρ)** | **R²_M*R²_Y*** | **R²_M~R²_Y~** |
| --- | --- | --- | --- |
| **Pre-hypertension** | 0.1 | 0.01 | 0.0066 |
| **Hypertension** | 0.3 | 0.09 | 0.0295 |
